# Recurrent late complications following congenital diaphragmatic hernia repair with prosthetic patches: a case series

**DOI:** 10.1186/1752-1947-3-7237

**Published:** 2009-05-26

**Authors:** Basil Bekdash, Baljit Singh, Kokila Lakhoo

**Affiliations:** 1 Department of Paediatric Surgery, John Radcliffe Hospital, Headington, Oxford, OX3 9DU, UK; 2 Nuffield Department of Surgery, John Radcliffe Hospital, Headington, Oxford, OX3 9DU, UK

## Abstract

**Introduction:**

Many different prosthetic materials have been used for repair of large posterolateral congenital diaphragmatic hernias, which cannot be primarily repaired. Almost 50% of patch repaired diaphragmatic hernias will recur. The ideal prosthetic material for congenital diaphragmatic hernia repair has yet to be established. We report on two cases with unusual (calcification) and late complications related to the prosthetic material used for diaphragmatic hernia repair.

**Case presentation:**

We report two cases of antenatally diagnosed left-sided diaphragmatic hernia that were repaired with a patch due to total absence of a diaphragm. Both Caucasian patients developed recurrent late graft complications. Case one was repaired on day 17 post-stabilisation and developed recurrence at 14½ months of age and again at 26 months of age. Case 2 underwent surgery on day 13 of life and developed recurrence at 4 months and again at 3 years of age. In the recurrent repairs, both synthetic and biomaterial patches were used. Both patients are well at long-term follow-up of 10 and 7 years, respectively.

**Conclusion:**

The ideal choice of patch material for diaphragmatic hernia repair remains a therapeutic challenge.

## Introduction

Congenital diaphragmatic hernias (CDH) occur with an estimated incidence of 1/2-4000 live births [1] and almost invariably require surgical intervention. The majority of defects are left-sided and sufficiently small to permit primary closure. Larger defects or total agenesis require closure with a prosthetic patch or flap, the latter usually for recurrent hernias. The patch material used may be the major contributing modifiable factor determining the incidence of late complications well after the time of surgery.

## Case presentation

### Case report 1

**Figure 1:**
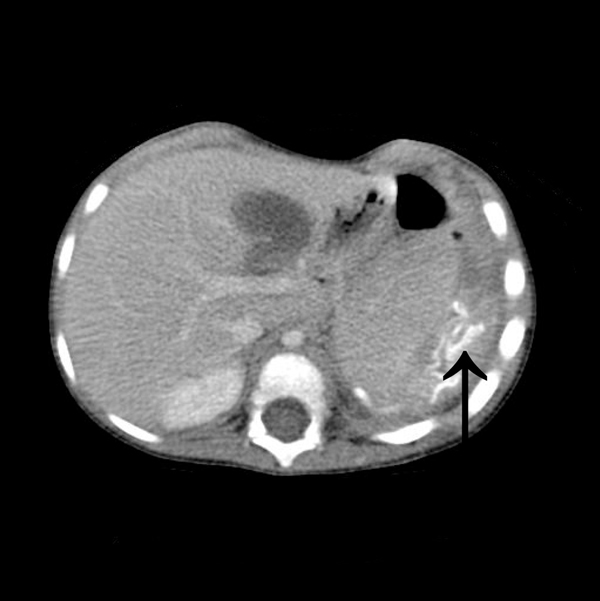
**Computed tomography demonstrating an abscess associated with a distorted radio-opaque Gore-Tex patch**. *S. pneumoniae* was isolated from the contents.

A full-term Caucasian female neonate with antenatally diagnosed left-sided CDH underwent surgical repair of a large defect at 17 days with Gore-Tex (expanded polytetrafluoroethene (PTFE); WL Gore & Associates Inc). One month following Nissen fundoplication and Stamm gastrostomy aged 14½ months, she presented with a recurrence through a ~2 cm anteromedial dehiscence of the patch. The defect was later closed primarily via thoracotomy and the hemidiaphragm reinforced with Surgisis (porcine small intestine submucosa (SIS) collagen; Cook Inc). At 26 months of age, the child presented with an abscess at the site of the previous thoracotomy and computed tomography demonstrated an intrathoracic collection associated with the prostheses (Figure 1). After initial drainage of the external component, at thoracotomy both collections were found to be in continuity and the distorted Gore-Tex patch within the abscess cavity was excised. There was sufficient fibrous 'neodiaphragm' to permit primary closure of the defect with no need for a further patch. To date, at 10 years of age there have been no further recurrences or complications.

### Case report 2

A full-term male Caucasian neonate with antenatally diagnosed left-sided CDH underwent surgical repair of a large defect on day 13 of life with a Permacol patch (porcine dermal collagen; Tissue Science Laboratories). A posterior recurrence was noted at 4 months of age and a posterolateral defect at the edge of the previous repair was closed with a Gore-Tex patch. At laparotomy, through the original subcostal incision, the Permacol derived tissue was noted to be fibrotic and calcified. Acute on chronic respiratory compromise heralded a further recurrence at 3 years of age confirmed by contrast enema. At laparotomy, the Gore-Tex patch was intact with little evidence of any residual Permacol and only a small anterior rim of diaphragm representing the original native tissue. The Gore-Tex patch was excised and the entire defect repaired with Surgisis. To date, at 7 years of age there have been no further recurrences or complications.

## Discussion

The repair of CDH has evolved over recent years with the development of patches composed of biomaterials reported to better integrate with host tissues and, eventually being replaced by them. The recent literature reports patch repair of CDH in up to 50% of cases [2] in contrast to earlier series where patch repair was less common [3] and may represent earlier referral and surgical intervention. These two cases highlight the problems related to the use of patches for CDH repair in individual patients.

Where primary closure is not possible, the goal of the repair is to provide a mechanically robust pleuroperitoneal partition that will endure for the patient's lifetime, potentially to normal life expectancy. As demonstrated in both cases, synthetic patch integrity is usually maintained during recurrence which largely occurs due to failure at the host-prosthesis interface. In contrast, failure of flap or biomaterial patch repair in the absence of infection (Case 2) occurs by patch failure, probably due to a combination of inadequate angiogenesis, host tissue in-growth and deterioration of the patch/flap due to atrophy or the inflammatory response.

Gore-Tex patches surpassed several similar materials in terms of clinical utility and there is good general surgical experience with their use in a variety of applications. Although durable and promoting a degree of angiogenesis, they remain *in situ* for life and are a potential site of microbial colonisation. There are usually few options following patch infection other than excision (Case 1) and in a recent series, 41% required revision within 3 years [4].

The two biomaterials used in these cases are collagen scaffolds derived from porcine tissue and marketed as acellular materials that are assimilated and eventually replaced by host tissue. Although collagen biomaterials meet European Union/Federal Drug Administration manufacturing and processing regulations, their acellularity has been questioned and the potential for zoonotic infections remains. Laboratory comparison of collagen-based and synthetic patches has produced inconsistent findings that are difficult to compare due to the diversity of experimental models utilised and combinations of materials studied. The former are generally found to produce higher fibroblast deposition. The latter induce a much greater inflammatory response with greater potential for adhesion formation. The reported mechanical properties are inconsistent [5].

This is the first time we have encountered calcification of a Permacol graft (Case 2) which may have reduced patch flexibility and contributed to the recurrence. Experimental evaluation of SIS derived collagen has shown that glutaraldehyde cross-linking promotes calcification, poor host-tissue incorporation and ultimately mechanical failure compared to peracetic acid treated and native SIS [6]. Perhaps significantly, commercially available Permacol is cross-linked whereas, currently, Surgisis is supplied in its native form. Problematic fibrotic reactions (orbital and urological surgery) and several instances of graft failure in orthopaedic applications have been reported but there is no systematic or comprehensive reporting of complications either in the literature or by the manufacturers. Experimental subcutaneous implantation [5,6] assesses a microenvironment more conducive to the angiogenesis necessary for successful assimilation in contrast to inherently less well vascularised joint spaces and the diaphragm where, in the absence of adhesions, host-tissue in-growth is limited to the patch periphery. Direct comparison of the angiogenic potential of various materials is hampered by difficulty in determining the proportion of the observed increase in vascularity due to angiogenesis and that due to the inflammatory response [5].

We experienced no early failure following use of Surgisis and in Case 1, the development of a fibrous membrane permitted primary closure without a further patch. Small series of successful use of Surgisis in diaphragmatic hernia repair have been reported [7] but not all cases are neonates/infants and mortality rates are high in such small groups.

Use of local muscle flaps (intercostals [8], reversed lentissimo dorsi [9]) for CDH repair has been reported but they produce significant body wall deformity and are largely restricted to recurrent CDH. Although the risk of infection is greatly reduced, denervated muscle tissue tends to atrophy predisposing it to further recurrence and attempts at microneural anastomosis have produced equivocal outcomes [9]. Lack of long-term follow-up and reluctance to publish ineffective approaches make evaluation difficult. Application of a non-deforming intraperitoneal (Toldt's) fascial flap has recently been reported but again it is too early to evaluate long-term outcomes [10].

## Conclusions

The literature presents series of variable size and clinical consistency but fails to provide a robust evidence base on which graft materials can be selected or approaches to CHD repair chosen with regard to long-term outcome. Recurrence produces major morbidity and the true lifetime incidence is not known. The medical literature lags behind the rapid development and modification of prosthetic materials and it is hoped that this situation will change in the near future.

## Abbreviations

CDH: congenital diaphragmatic hernias; PTFE: polytetrafluoroethene.

## Consent

Written informed consent was obtained from the patients for publication of this case series and any accompanying images. A copy of the written consent is available for review by the Editor-in-Chief of this journal.

## Competing interests

The authors declare that they have no competing interests.

## Author contributions

BB and BS prepared the manuscript and did the literature search. KL conceived the idea, managed the patients, assisted with the literature search, and reviewed the manuscript.
